# The complete mitochondrial genome of *Epomophorus gambianus* (Chiroptera: Pteropodidae) and its phylogenetic analysis

**DOI:** 10.1080/23802359.2016.1181993

**Published:** 2016-07-08

**Authors:** Silke A. Riesle-Sbarbaro, Stefan P. W. de Vries, Samuel Stubbs, Kofi Amponsah-Mensah, Andrew A. Cunnigham, James L. N. Wood, David R. Sargan

**Affiliations:** a Department of Veterinary Medicine, University of Cambridge, Cambridge, UK;; b Zoological Society of London, Institute of Zoology, London, UK;; c Department of African Center for Wetlands Conservation, University of Legon, Accra, Ghana

**Keywords:** Chiroptera, *Epomophorus gambianus*, mitogenome, phylogeny

## Abstract

The Gambian epauletted fruit bat, *Epomophorus gambianus*, is widely distributed across sub-Saharan Africa. Its assembled and annotated mitochondrial genome (GenBank accession no. KT963027) is 16,702 bases in length, containing 13 protein-coding genes, 22 transfer RNA genes, two ribosomal RNA genes and two non-coding regions: the control region (D-loop) and the origin of light-strand replication (O_L_). The average base composition is 32.2% A; 27.6% C; 14% G; and 26.1% T. The mitogenome presented a structural composition greatly conserved between members of the Pteropodidae family.

*Epomophorus gambianus* is an old world fruit bat, part of one of the most diversified taxa: the family Pteropodidae (Simmons 2005). Throughout its distribution, *Epomophorus* co-roost with several Pteropodid bats; including members of Rousettini, Myonycterini and Epomorphorini tribes (Nesi et al. 2013). With increasing number of Chiropteran studies exploring their role as disease reservoirs (Calisher et al. 2006; Baker et al. 2012), it is imperative to resolve their evolutionary relationships accurately.

Using the DNeasy Blood and Tissue Kit (Qiagen, Valencia, CA), DNA was extracted from wing membrane biopsies from five *E. gambianus* captured in Ghana (6°59′32.12″N; 0°25′32.12″E) and stored in the University of Cambridge, UK. A DNA sequencing library was constructed and sequenced on an Illumina MiSeq (Supplementary methods). A total of 27,143,463 paired-end reads were mapped with CLC Genomics Workbench v7.5.1 software (CLC Bio, Aarhus, Denmark) to *Rousettus aegyptiacus* (GenBank AB205183.1). The resulting assembly (16,624 bp) was used as reference for *de novo* assembly, presenting 98% coverage against the reference mitochondrion. Decreasing coverage was observed downstream of bp position 15,900. The lowest coverage (< 7*x*) was found within D-loop, in a region that overlapped with a perfect repeat (CATACACGTACG)_23_. Sanger sequencing was used for gap closure (Supplementary Table 1); the Sanger sequences were mapped to the assembly using GENEious v8.1 (CLC Bio, Aarhus, Denmark). The resulting assembly was annotated by referencing nine closely related mt-genomes (Supplementary Table 2).

Most of the genes are encoded on the heavy strand of the mitogenome with the exception of eight tRNA and ND6 protein-coding gene that are on the light strand. Except for NADH dehydrogenase 1, 2, 3 and 5, ATG was the starting codon. TAA was the stop codon except for gene *ND2* ending with TAG, and *Cyt b* with AGG. *ND1*, *COX III* and *ND4* presented incomplete stop codons (Supplementary Table 3).

To evaluate the evolutionary relationship of *E. gambianus* to other Chiroptera, eighteen mitogenomes were aligned using Mega6 (Tamura et al. 2013). Three methods were used to produce phylogenetic trees and the resulting topologies were visualized with FigTree v1.4.2 (CLC Bio, Aarhus, Denmark). Node supports were assembled in a consensus tree with TreeGraph 2.4.0-456 beta (Stover & Muller 2010) and manually verified and merged in Adobe Illustrator (Figure 1).

**Figure 1.**
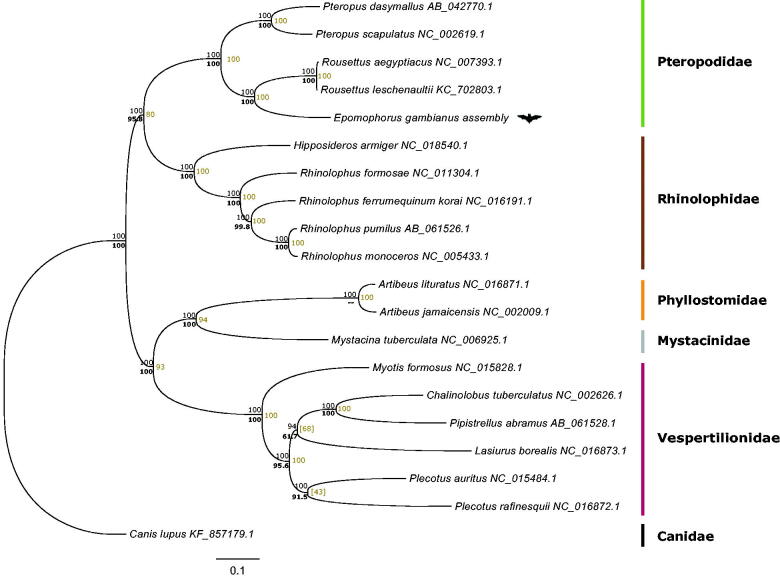
Phylogenetic tree of complete bat mitogenomes. Three methods are shown: maximum parsimony (MP) created using a Min-mini heuristic algorithm and 1000 bootstrap replicates; maximum-likelihood (ML) constructed in PAUP 4.0 (Swofford 2011) with bootstrap percentages computed after 1000 replicates using PhyML software (Guindon et al. 2010); and Bayesian Inference performed in MrBayes 3.1.2 (Huelsenbeck & Ronquist 2001) using MCMC algorithm, running six chains for 300,000 generations and discarding the first 25% of trees. The best-fit nucleotide substitution model for ML and Bayesian methods were selected with JmodelTest 2.1.7 (Darriba et al. 2012) and PAUP 4.0 beta software. Bayesian posterior probabilities appear above and left to the node it supports. Bootstrap values appear below in bold text for ML and in yellow for MP, values inside square brackets refer to conflicting topology. *Epomophorus gambianus* is denoted with a bat illustration. *Canis lupus* complete mt-genome was assigned as an out-group.

The presented assembly is in agreement with previous classification of the species and corroborate the Yinpterochiroptera/Yangochiroptera division (Teeling et al. 2005; Nesi et al. 2013). *Epomophorus gambianus* mitogenome could prove useful for resolving introgressive hybridizations, a phenomenon that has been recorded between this species and the sympatric *Micropteropus pusillus* (Nesi et al. 2011).
