# Corrigendum

**DOI:** 10.1080/23802359.2017.1317133

**Published:** 2017-04-21

Silke A. Riesle-Sbarbaro, Stefan P. W. de Vries, Samuel Stubbs, Kofi Amponsah-Mensah, Andrew A. Cunnigham, James L. N. Wood, David R. Sargan. 2016. The complete mitochondrial genome of Epomophorus gambianus (Chiroptera: Pteropodidae) and its phylogenetic analysis. Mitochondrial DNA Part B Resour. 1:447-449.

http://dx.doi.org/10.1080/23802359.2016.1181993

When the above article was first printed and published online, the author name Andrew A. Cunnigham was spelt incorrectly. The name should read as Andrew A. Cunningham.

